# Pancreatitis and myocarditis followed by pulmonary hemorrhage, a rare presentation of leptospirosis- A case report and literature survey

**DOI:** 10.1186/1471-2334-13-38

**Published:** 2013-01-24

**Authors:** Nuwan Ranawaka, Vijayabala Jeevagan, Panduka Karunanayake, Saroj Jayasinghe

**Affiliations:** 1 University Medical Unit, National Hospital of Sri Lanka, Colombo, 10, 01000, Sri Lanka; 2 Department of Medicine, Faculty of Medicine, University of Colombo, Colombo, 08, 00800, Sri Lanka

**Keywords:** Leptospirosis, Pancreatitis, Myocarditis, Pulmonary hemorrhage, Toll like receptors

## Abstract

**Background:**

Leptospirosis is a potentially fatal disease which can cause multi-organ dysfunction. It can rarely present as acute pancreatitis. This is the first ever report of leptospirosis presenting with acute pancreatitis and myocarditis followed by diffuse pulmonary hemorrhages to the best of our knowledge.

**Case presentation:**

A 15-year-old South Asian boy presented with high grade fever, epigastric discomfort and was anicteric on admission. He developed tachycardia, transient hypotension, changes of electro-cardiogram and positive troponin I suggestive of myocarditis. Acute pancreatitis was diagnosed with 12 fold high serum amylase and with the evidence of computerized tomography. Then he developed diffuse pulmonary hemorrhages and later acute renal failure. Leptospirosis was confirmed by positive leptospira IgM, negative IgG and strongly positive Microscopic Agglutination Test. Other possible infective and autoimmune causes were excluded. Patient recovered completely with antibiotics and the supportive care.

**Conclusion:**

This case illustrates diagnostic difficulties especially in resource poor settings where leptospirosis is common. Additionally it highlights the fact that leptospirosis should be considered in patients presenting with pancreatitis which can be complicated with myocarditis and diffuse pulmonary hemorrhages. We hypothesize that Toll like receptors may play a role in such systemic involvement.

## Background

Leptospirosis is a potentially fatal re-emerging disease globally [1]. It is known to cause multi organ dysfunction including myocarditis, diffuse pulmonary hemorrhage and very rarely pancreatitis [2,3]. We report an unusual presentation of leptospirosis with initial myocarditis and pancreatitis progressing in to pulmonary hemorrhages along with other complications which led to a diagnostic dilemma. This is the first ever report of such a presentation in leptospirosis to the best of our knowledge. Importantly the patient recovered dramatically with antibiotics and supportive care.

## Case presentation

A 15-year-old previously healthy Sri Lankan boy from an urban crowded area, presented with high grade intermittent fever for 5 days with epigastric discomfort and backache without severe myalgia. He had neither clinically detectable focus of infection nor bleeding manifestations. He had not traveled to endemic areas of malaria or typhus. He had not consumed drugs, alcohol or indigenous medications.

On admission he was anicteric, pink, well hydrated and temperature was 38.8°C. There was tachycardia around 150 beats/min and an initial blood pressure of 110/70 mmHg which dropped down to 80/60 mmHg after 8 hrs. Tachypnoea was the only abnormality detected on the examination of respiratory system while the precordial examination was normal. There was mild epigastric discomfort on palpation but guarding, rigidity and organomegaly were not detected.

The electrocardiogram demonstrated sinus tachycardia with ST segment depressions and troponin I was positive. The transthoracic echocardiography revealed an ejection fraction of 60%, normal diastolic function and chamber sizes. Myocarditis was considered and normal blood pressure was achieved within 1 hour with inotropic support [4,5].

On admission biochemistry revealed serum amylase of 4200 IU/L (70–340) and normal renal functions. Ionized calcium was low [0.9 mmol/l (1.1-1.4)]. Non-contrast CT abdomen demonstrated prominent, edematous pancreas suggesting pancreatitis, along with hepatomegaly.

**Figure 1:**
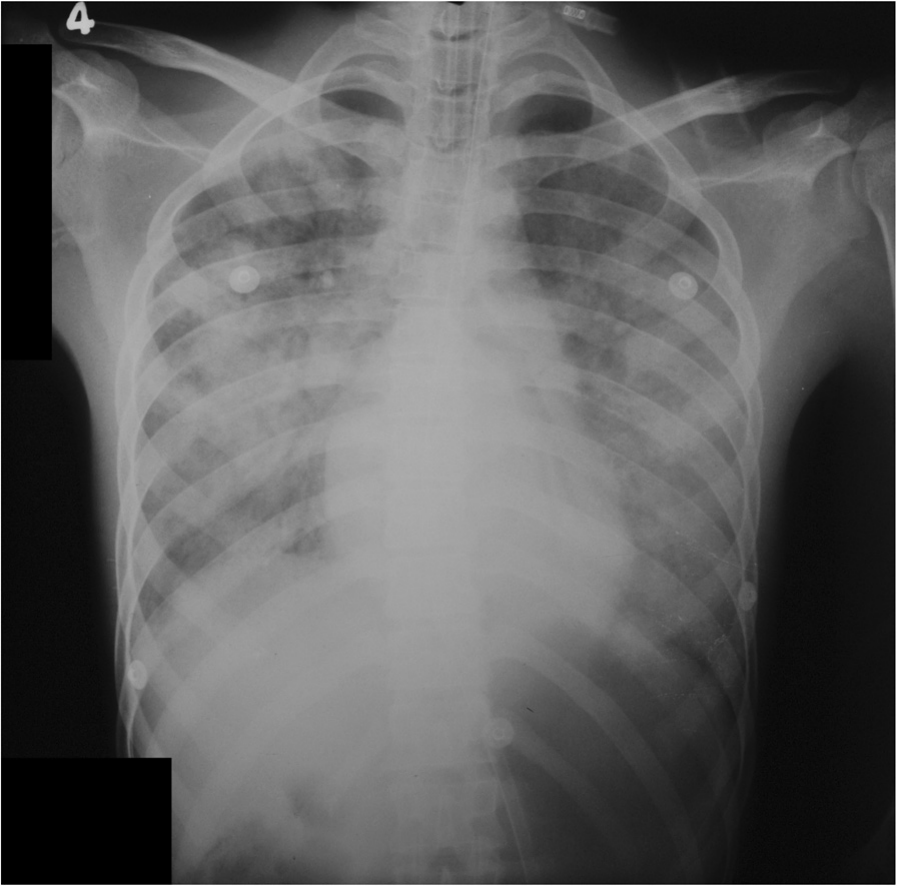
Chest x ray of the patient demonstrating diffuse pulmonary hemorrhages.

The patient developed gradually worsening dyspnea with severe hemoptysis and hypoxia necessitating endotracheal intubation and ventilator support on the third hospitalized day. Frank blood was aspirated from the endotracheal tube. Repeat chest x ray demonstrated newly developed bilateral diffuse alveolar shadows suggesting diffuse pulmonary hemorrhages (Figure 1). He was hemodynamically stable and maintaining normal blood pressure without inotropic support while repeat echocardiography was normal. Central venous pressure(CVP) was 10 cm H2O.

Simultaneously his renal functions deteriorated with an elevation of serum creatinine upto 521 μmol/l (60–120). The patient was not oliguric during the first 2 days despite the initial episode of transient hypotension which developed 8 hrs after the admission. After recovering from the initial hypotensive episode within 1 hr, he was normotensive, normovolemic with normal CVP throughout the hospital stay. During the 3rd day he gradually became oliguric with rising serum creatinine level. Urinalysis revealed proteinuria of 300 mg/dl and hematuria of 100 per high power field without dysmorphic red cells or casts. Towards the latter part of the 3rd day he underwent hemodialysis. Hemodialysis was offered twice to the patient until the stabilization of renal function and then urine output and the serum creatinine was gradually normalized.

Icterus was detected along with the deterioration of renal function. Total and direct bilirubin was elevated [184 μmol/l (5–21) and 76 μmol/l (<3.4) respectively] and increased aspartate transaminase, alanine transaminase and alkaline phosphatase [96 units/l (10–35), 141 units/l(10–40) and 685 units/l(100–360) respectively] was detected. Creatine phospho kinase was high [590 units/l (25–174)].

The hemoglobin level dropped from 13 g/dl to 8 g/dl with the pulmonary hemorrhage and the platelet count dropped from 125000 to 20000/mm3. Total leucocyte count was normal (7000/mm3) with 70% neutrophils with toxic changes. There were no polychromasia or fragmented erythrocytes in blood picture. International normalized ratio was 1.6 while activated partial thromboplastin time was normal.

With the progression of the clinical course, multisystem involvement with initial pancreatitis and possible myocarditis followed by pulmonary hemorrhage was unusual and led to a diagnostic dilemma in view of the aetiology. Later, along with the renal and hepatic involvement, the detection of positive leptospira IgM, negative IgG and a titer of 3200 in microscopic agglutination test (MAT) for leptospirosis enlightened the clinical picture. Negative hepatitis serology, dengue NS1 antigen and antibodies, serology for mycoplasma pneumonia excluded the other possible infective pathologies. Anti-nuclear antibodies, anti-neutrophil cytoplasmic antibodies (MPO and PR3) were negative and complement levels were normal.

Intravenous meropenem 500 mg 8 hourly and benzyl penicillin 2,000,000 units 6 hourly was started empirically and continued for 2 weeks along with supportive care. Ventilator support was offered for 4 days and hemodialysis was performed twice. Patient improved dramatically, and became asymptomatic. Normal levels of serum amylase, renal function, cardio-respiratory functions, transaminases and hematological parameters were achieved by the end of 2nd week.

## Discussion

Leptospirosis is a zoonotic disease caused by *Leptospira interrogans* complex leading to multi-system involvement [2]. Pathogenesis of organ dysfunction is yet to be fully understood. It is thought to be related to leptospira burden, associated cytotoxic factors in the tissue especially in liver and kidney and host immune mechanism especially in lungs [2,6,7]. Recently it was found that the linear deposition of immunoglobulins and complements on the alveoli may play a role in pulmonary haemorrhages [7]. Highly sensitive and specific MAT is the gold standard serological test for the diagnosis which became strongly positive repeatedly in this patient [2]. This case highlights an unusual presentation of leptospirosis with involvement of heart and pancreas followed by diffuse pulmonary hemorrhages.

Acute pancreatitis is a very rare manifestation in leptospirosis and PUBMED search using criteria of “Pancreatitis” and “Leptospirosis” revealed only 21 articles which were published in English literature to date. Pancreatitis on presentation was recognized in few articles [3,8-13]. Pulmonary hemorrhage in combination with pancreatitis in leptospirosis was reported only once previously by Daher Ede F. et al.(2003), yet it was not known at which point of clinical course the pancreatitis was developed [14]. Thus this case is the first ever report of leptospirosis presenting with acute pancreatitis along with cardiac involvement, complicated with pulmonary hemorrhages.

**Figure 2:**
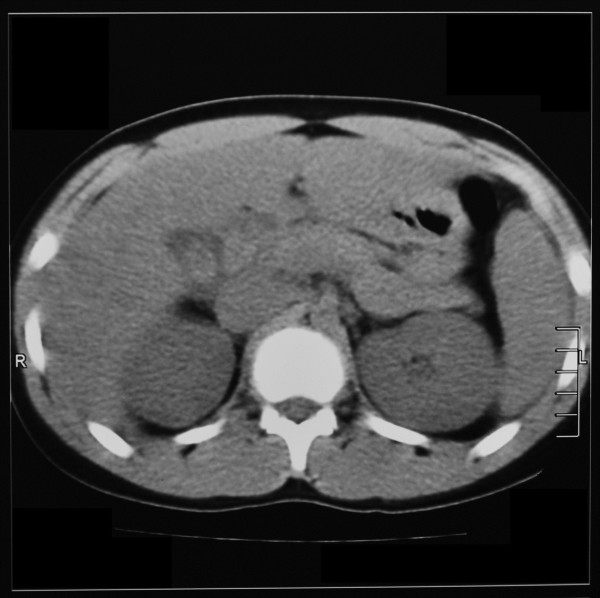
Non-contrast CT Abdomen of the patient demonstrating prominent edematous pancreas, pericholicysitc fluid along with hepatomegaly.

The diagnosis of pancreatitis was based on biochemical and radiological evidence (Figure 2). Increased serum amylase more than 12 times the upper normal value is highly specific for pancreatitis in this patient especially at a time of normal renal functions [15,16]. Though serum lipase was not available, low serum calcium and CT evidence of mild pancreatitis supported the diagnosis [15]. Concomitant myocarditis was suspected with clinical evidence of tachycardia, transient hypotension and ST segment changes in ECG with positive troponin I despite normal echocardiography [4,5,17]. Diffuse pulmonary hemorrhage which is well known in leptospirosis, was suggested by the presence of frank endotracheal bleeding, respiratory compromise and typical chest x-ray features along with dropping hematocrit in the absence of other source of bleeding [18-20]. Proteinuria and hematuria suggested an intra-renal pathology as the aetiology of renal failure. Further, normal CVP at the onset of renal failure ruled out the possibility of hypovolemia or 3rd space loss in pancreatitis being the aetiology.

The other endemic pathogens which lead to multisystem involvement such as dengue hemorrhagic fever, hepatitis virus, typhus and mycoplasma were excluded. Autoimmune multi-system disorders such as systemic lupus erythematosus, vasculitis were also differentiated.

An immunological basis for pathogenesis of leptospirosis including Toll like receptor (TLR) 2 activation is described recently [6]. TLR 2 plays a major role in the development of pulmonary and renal manifestations of leptospirosis [21,22]. Leptospira lipoprotein LipL32 triggers an inflammatory response in renal proximal tubule cells by activation of TLR 2 and hence nuclear factor-[kappa]B and mitogen-activated protein kinases [6,22]. Increased expression of TLR 2 was detected in the pulmonary vasculature in diffuse pulmonary hemorrhages of leptospirosis [21]. Though it is not yet described in relation to leptospirosis, TLR may contribute to myocarditis in sepsis and may involve in the pathogenesis of acute pancreatitis [23,24]. Bacterial peptidoglycan associated lipoprotein uses the TLR2 signaling pathway to induce cardio-myocyte dysfunction and inflammatory response in mice [23]. In acute pancreatitis, increased expression and activation of TLR 2/4 has been recognized and their role in multi-organ involvement was identified [24]. Thus a similar mechanism involving TLR may explain the presentation of our patient.

Though the reported incidence of pancreatitis in leptospirosis is infrequent, in reality pancreatic involvement may be more common. Under-recognition could be due to several reasons. Pancreatic involvement could be subclinical or clinically unrecognized when dramatic and rapidly dynamic alterations of clinical and biochemical parameters take place in multi-organ dysfunction in leptospirosis. Thus a clinician may find it difficult to identify each and every complication such as pancreatitis, acalculous cholecystitis, cerebral venous thrombosis and myositis [25,26].

Additionally in a clinical setup, when a patient presents with acute pancreatitis alone as in this case, leptospirosis might not be considered as an aetiology in the initial work-up because of the rarity. Later the patient may develop multi-organ failure due to leptospirosis, yet that might be attributed to the multi-organ involvement of acute pancreatitis. Ultimately, recognition of leptospirosis might get delayed compromising the optimum management.

## Conclusion

This case illustrates diagnostic difficulties especially in resource poor settings where leptospirosis is more common. Pulmonary hemorrhage with pancreatitis in leptospirosis was reported only once before and this is the first occasion in leptospirosis which reports pancreatitis and myocarditis leading to pulmonary hemorrhage. We hypothesize an immunological basis involving TLR for such presentation. In conclusion, leptospirosis should be considered in patients presenting with pancreatitis which can be complicated with myocarditis and diffuse pulmonary hemorrhages. It should not be mistaken as idiopathic pancreatitis though the clinical presentation and complications would be the same.

## Consent

Written informed consent was obtained from the patient and the parent for publication of this case report and accompanying images. A copy of the written consent is available for review by the Series Editor of this journal.

## Abbreviations

CT: Computerized tomogram; MAT: Microscopic agglutination test; NS 1: Nonstructural protein 1; MPO: myeloperoxidase; PR3: Proteinase 3; ECG: Electrocardiogram; TLR: Toll like receptor.

## Competing interests

The authors declare that they have no competing interests.

## Authors’ contribution

All authors were involved in the management of the patient. NR and VJ researched the background literature on the case and wrote the first draft. PK and SJ contributed towards the discussions and analysis of the case. All authors read and approved the final manuscript.

## Authors’ information

NR(MBBS) and VJ(MBBS) are registrars in internal medicine in the University Medical Unit, National Hospital, Colombo, Sri Lanka. PK(MBBS, MD, MRCP) is a Senior Lecturer in Medicine and Consultant Physician, and SJ(MBBS, MD, FRCP, FCCP, MD Bristol) is Professor in Medicine, in the Department of Clinical Medicine, Faculty of Medicine, University of Colombo, Sri Lanka.

## Pre-publication history

The pre-publication history for this paper can be accessed here:

http://www.biomedcentral.com/1471-2334/13/38/prepub
